# Block Diagonal Hybrid Precoding and Power Allocation for QoS-Aware BDMA Downlink Transmissions

**DOI:** 10.3390/s20164497

**Published:** 2020-08-11

**Authors:** Guanchong Niu, Qi Cao, Manon Pun

**Affiliations:** 1 School of Science and Engineering, The Chinese University of Hong Kong (Shenzhen), Shenzhen 518172, China; 216019007@link.cuhk.edu.cn (G.N.); Caoqi@cuhk.edu.cn (Q.C.); 2 Shenzhen Research Institute of Big Data, Shenzhen 518172, China; 3 Shenzhen Key Laboratory of IoT Intelligent Systems and Wireless Network Technology, Shenzhen 518172, China

**Keywords:** BDMA, hybrid beamforming, block diagonal precoder, power allocation

## Abstract

Beam Division Multiple Access (BDMA) with hybrid precoding has recently been proposed for multi-user multiple-input multiple-output (MU-MIMO) systems by simultaneously transmitting multiple digitally precoded users’ data-streams via different beams. In contrast to most existing works that assume the number of radio frequency (RF) chains must be greater than or equal to that of data-streams, this work proposes a novel BDMA downlink system by first grouping transmitting data-streams before digitally precoding data group by group. To fully harvest the benefits of this new architecture, a greedy user grouping algorithm is devised to minimize the inter-group interference while two digital precoding approaches are developed to suppress the intra-group interference by maximizing the signal-to-interference-and-noise ratio (SINR) and the signal-to-leakage-and-noise ratio (SLNR), respectively. As a result, the proposed BDMA system requires less RF chains than the total number of transmit data-streams. Furthermore, we optimize the power allocation to satisfy each user’s quality of service (QoS) requirement using the D.C. (difference of convex functions) programming technique. Simulation results confirm the effectiveness of the proposed scheme.

## 1. Introduction

To meet the explosive demand for higher user data rates, it is envisioned that the next-generation cellular systems will be equipped with massive antenna arrays. Capitalizing on a large number of antennas at the base station (BS), Beam Division Multiple Access (BDMA) has recently been proposed as a promising method for 5G communications [1,2,3,4]. Different beams are allowed to transmit multiple users’ data-streams from BS. In contrast to the more conventional multiple access schemes such as Code Division Multiple Access (CDMA), Time Division Multiple Access (TDMA) or Orthogonal Frequency Multiple Division Access (OFDMA) that multiplex users in code, time and frequency domains, BDMA separates users in the beam space by transmitting data to different users in orthogonal beam directions. BDMA was first proposed in [1] to decompose the multi-user multiple-input multiple-output (MU-MIMO) system into multiple single-user MIMO channels by multiplexing multiple users’ data onto non-overlapping beams. Since beamforming is commonly implemented in the analog domain using low-cost phase shifters, BDMA becomes particularly attractive in practice in recent years. Moreover, joint beam selection and user scheduling were formulated under the Lyapunov-drift optimization framework before the optimal user-beam scheduling policy for BDMA was derived in a closed-form [2]. However, the assumption of non-overlapping orthogonal beams is generally difficult to be satisfied in practice. As a result, analog-only BDMA applications are heavily handicapped by the non-orthogonal inter-user interference among beams.

In the meantime, digital precoding has been widely investigated as an effective signal processing method to suppress the inter-user interference for MU-MIMO. It is well known that the classical fully digital precoding requires a dedicated radio frequency (RF) chain for each antenna. However, power consumption and the high hardware cost render the fully digital precoding impractical for massive MIMO systems [5,6,7]. To address this challenge, hybrid digital and analog beamforming has been proposed for massive MIMO transmissions by separating the precoding process into two steps, namely analog and digital precoding [8,9]. More specifically, the transmitted signals are first precoded digitally using a smaller number of RF chains followed by the analog precoder exploited by a much larger number of low-cost phase shifters [10,11]. As a result, the hybrid analog-digital precoding architecture requires significantly fewer RF chains as compared to the fully digital precoding [12]. It has been reported in the literature that the hybrid beamforming structure is capable of achieving performance compared to the fully digital beamforming scheme if the number of RF chains at each end is greater than or equal to twice the number of the data-streams [13]. Therefore, the hybrid precoded massive MU-MIMO system can benefit from the interference suppression supplied by the digital precoding while harvesting large antenna beamforming gains by implementing the massive antennas available in the systems [14]. This hybrid structure is particularly attractive for millimeter wave (mmWave) MIMO systems to support the transmissions of Gbps-order data throughput by exploiting the vast vacant spectrum available at RF of 6 GHz or above [15]. Furthermore, the notion of block diagonal (BD) precoding was first introduced to the conventional fully digital schemes to reduce the precoding complexity in [16]. By dividing the inverse of a large matrix into the inverse of multiple much smaller matrices, the BD precoding can be efficiently exploited with only marginal or no performance degradation as compared to the fully digital precoding [16]. In recent years, the BD design has been extended to the hybrid precoding for MU-MIMO [17]. However, most existing hybrid BD precoding schemes were constructed based on a crucial assumption, i.e., the number of RF chains must be no less than the total number of data-streams to be transmitted. Some pioneering works have investigated to relax this limitation by implementing the state-of-the-art fast-speed phase shifters and switches that can change their states symbol by symbol [18]. However, [19] requires users to resume their symbols via the compressive sensing technique, which makes the scheme impractical for low-complexity receivers.

Meanwhile, power allocation is also an important problem in co-channel interference management for multi-user wireless networks. In many MIMO applications, it is desirable to design a system satisfying the quality of service (QoS) constraint for each user by adjusting the power allocated to different users [20]. Since the objective function is highly non-convex, the problem is usually very difficult and complicated, especially for the coupled analog and digital precoding constraints [21]. Therefore, most existing works maximize the sum-rate capacity by implementing the water-filling algorithm without considering the QoS requirement for each user. For instance, [22] alternatively optimized the power allocation for sum-rate maximization by using the water-filling algorithm, assuming that the analog precoders are strictly orthogonal among distinct users. However, the water-filling algorithm cannot satisfy the per-user QoS constraint as users with poor channel conditions are not allocated any transmit power by the water-filling algorithm. To cope with this problem, the signal-to-interference-and-noise ratio (SINR)-balanced power allocation has been proposed to achieve identical SINR for all users [23,24,25,26]. However, the system performance using the SINR-based power allocation is limited by the user with the worst channel conditions.

In this work, we propose a downlink BDMA scheme empowered by BD digital precoding and global power allocation over multipath channels. Compared to the existing BDMA works [1,2], our proposed BDMA schemes can substantially suppress multi-user interference without requiring perfectly orthogonal beams as residual interference can be greatly removed by digital precoding. Furthermore, in sharp contrast to the conventional hybrid precoding schemes, the proposed scheme can use fewer RF chains than the number of transmit data-streams by exploiting the hybrid BD precoding architecture built upon the state-of-the-art fast-speed phase shifters [27] and switches [18]. Furthermore, an iterative algorithm for power allocation is proposed to satisfy per-user QoS requirement based on the difference of convex functions (D.C.) programming technique.

The main contributions of this paper are summarized as follows:

- A block diagonal hybrid precoding scheme is proposed by exploiting the state-of-the-art fast-speed phase shifters and switches. The resulting scheme can use fewer RF chains than the number of transmit data-streams by jointly performing hybrid analog-digital precoding and user-beam grouping.
- Furthermore, we develop a greedy grouping algorithm to minimize the inter-group interference while maximizing the intra-group interference. Then, the intra-group interference is eliminated by two proposed digital precoders, namely the SINR- and SLNR-based precoders.
- In contrast to most works in the literature that used the single-path channel model, we analyze the sum-rate capacity using the multipath channel model.
- Finally, for given analog and digital precoders, an optimized power allocation scheme is derived to satisfy per-user QoS requirement by using the D.C. programming technique.

The rest of this paper is organized as follows: In Section 2, we present the block diagonal system model with reduced RF chains before formulating the optimization problem. By allocating the power uniformly to each user, the analog and digital precoders are derived in Section 3. After that, the performance of the proposed system is analyzed in Section 5. Finally, to satisfy the QoS constraint, Section 6 proposes a QoS-aware power allocation algorithm based on the D.C. programming technique followed by extensive numerical results presented in Section 7.

Notation: In this paper, we use uppercase boldface and lowercase boldface letters to denote matrices and vectors, respectively. $I_{N}$ denotes the identity matrix with size N×N. $A^{T}$ and $A^{H}$ denote the transpose and conjugate transpose of A, respectively. $A^{†}$ is the pseudo inverse of A while ∥A∥ stands for the L2 norm of A and |A| denotes the absolute value of *A*. ${\left[a\right]}_{i}$ denotes the *i*-th element of a. |I| is the cardinality of the enclosed set I. $X^{2}\left(k\right)$ represents the chi-square distribution with *k* degrees of freedom. 〈A,B〉 is the inner product of A and B. X←x stands for the addition of element *x* to set X while X∖x removal of element *x* from X. Finally, E[·] denotes the expectation of the enclosed random variable.

## 2. System Model and Problem Formulation

**Figure 1:**
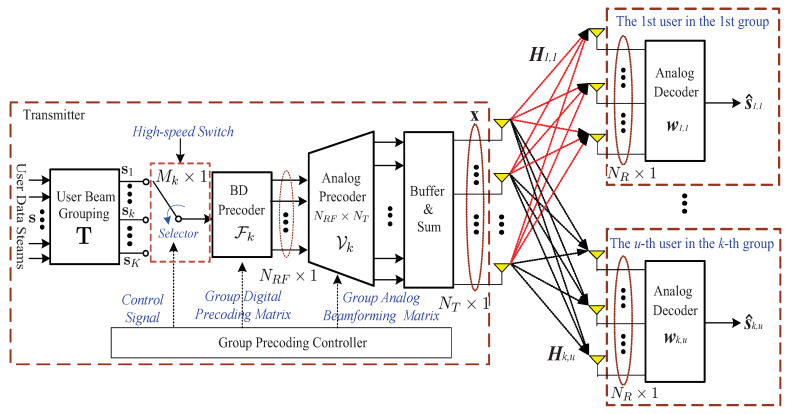
Block diagram of the hybrid precoding system under consideration.

In this paper, we consider a MU-MIMO downlink system as shown in Figure 1 in which $N_{U}$ users are scheduled for service. $N_{RF}$ RF chains and $N_{T}$ antennas are equipped on BS which transmits $N_{U}$ data-streams to $N_{U}$ receivers with $N_{R}$ receive antennas at each time slot. In a practical MIMO system, the number of RF chains is typically much smaller than the number of antennas, i.e., $N_{RF}\ll N_{T}$. We assume that only one data stream is designated to each scheduled receiver for transmission. Denoted by s(n) the *n*-th block of $N_{U}$ data to be transmitted, s(n) has unit power with $E\left[{\mathrm{ss}}^{H}\right]=\frac{1}{N_{U}}I_{N_{U}}$. In the sequel, we can concentrate on a single block and omit the temporal index *n* for notation simplicity.

### 2.1. Transmitter

In our proposed group-by-group BD digital precoding system shown in Figure 1, the $N_{U}$ users are first divided into *K* groups with the group size being $M_{K}$, where $0<M_{k}\leq N_{U}$ for k=1,2,⋯,K. It is clear that $\sum_{k=1}^{K}M_{k}=N_{U}$. Accordingly, the data-streams s can be rewritten in groups as:

(1) $$s={\left[s_{1}^{T},s_{2}^{T},\cdots ,s_{K}^{T}\right]}^{T},$$

where $s_{k}\in C^{M_{k}\times 1}$ is the data vector transmitted to the users in the *k*-th group and modeled as:

(2) $$s_{k}={\left[s_{k,1},s_{k,2},\cdots ,s_{k,M_{k}}\right]}^{T},$$

with $s_{k,u}$ being the data transmitted to the *u*-th user in the *k*-th group for $u=1,2,\cdots ,M_{k}$.

Next, we focus on modeling the digital precoding process. Denoted by $F_{k}$ of $N_{RF}\times M_{k}$ the digital precoder for the *k*-th group for k=1,2,⋯,K, $F_{k}$ can be written as:

(3) $$F_{k}=\left[f_{k,1},f_{k,2},\cdots ,f_{k,M_{k}}\right],$$

where $f_{k,u}$ represents the digital precoding vector for the *u*-th user in the *k*-th group. Thus, the overall digital precoding matrix can be expressed as a block diagonal matrix as follows:

(4) $$F=\left[\begin{array}{cccc} F_{1} & \cdots & 0 & 0\\ \vdots & F_{2} & \vdots & \vdots \\ 0 & \cdots & \ddots & 0\\ 0 & \cdots & 0 & F_{K} \end{array}\right].$$

Clearly, inverting a BD matrix is less computationally expensive than a non-BD matrix of the same dimension. Therefore, the BD structure of F in Equation (4) can potentially lead to reduced computational complexity.

Similarly, we model the corresponding analog precoder in groups as

(5) $$V=\left[V_{1},V_{2},\cdots ,V_{K}\right],$$

where $V_{k}$ of $N_{T}\times N_{RF}$, the analog precoder for the *k*-th group for k=1,2,⋯,K, is given by:

(6) $$V_{k}=\left[v_{k,1},v_{k,2},\cdots ,v_{k,M_{k}}\right],$$

with $v_{k,u}$ being the analog beamforming vector for the *u*-th user in the *k*-th group.

Finally, the resulting hybrid precoded signal $x\in C^{N_{T}\times 1}$ is transmitted to all $N_{U}$ users.

(7) $$x=V\cdot F\cdot s=\sum_{k=1}^{K}V_{k}F_{k}s_{k}.$$

### 2.2. Channel Models

We denote $H_{k,u}\in C^{N_{R}\times N_{T}}$ the MIMO multipath channel matrix between the transmitter and the *u*-th receiver in the *k*-th group using the Saleh-Valenzuela model [8]:

(8) $$H_{k,u}=D_{k,u}\sum_{l=1}^{L_{k,u}}\alpha_{k,u,l}a_{R}\left(\phi_{k,u,l}^{r},\theta_{k,u,l}^{r}\right)a_{T}^{H}\left(\phi_{k,u,l}^{t},\theta_{k,u,l}^{t}\right),$$

where $L_{k,u}$ is the total number of multipath components between the transmitter and the *u*-th user in the *k*-th group. Furthermore, $\alpha_{k,u,l}$, $\theta_{k,u,l}^{r}/\phi_{k,u,l}^{r}$ and $\theta_{k,u,l}^{t}/\phi_{k,u,l}^{t}$ are the complex path gain, angles of arrival (AoA) and azimuth/elevation angles of departure (AoD) of the *l*-th path of the *u*-th user in the *k*-th group, respectively. Furthermore, a(ϕ,θ) is the array response vector. Finally, $D_{k,u}=\sqrt{\frac{N_{T}N_{R}}{L_{k,u}}}$ is a constant parameter. For a uniform planar array (UPA) of size P×Q considered in this work, the array response vector is given by [8]:

(9) $$a\left(\phi ,\theta \right)=\frac{1}{\sqrt{PQ}}\left[1,e^{j\kappa d(\sin\phi \sin\theta +\cos\theta )},\right.{\left.\cdots ,e^{j\kappa d\left((P-1)\sin\phi \sin\theta +(Q-1)\cos\theta \right)}\right]}^{T},$$

where $\kappa =\frac{2\pi }{\lambda }$ is the wavenumber and *d* is the distance between two adjacent antennas.

### 2.3. Receiver

Consequently, we formulate the receiver structure of the *u*-th user in the *k*-th group. The received signal is represented by

(10) $$\begin{array}{c} y_{k,u}=\underset{\mathrm{Desired}\;\mathrm{Signal}}{\underset{︸}{H_{k,u}V_{k}f_{k,u}s_{k,u}}}+\underset{\text{Intra-group Interference}}{\underset{︸}{H_{k,u}V_{k}\sum_{\begin{array}{c} i=1\\ i\neq u \end{array}}^{M_{k}}f_{k,i}s_{k,i}}}+\underset{\text{Inter-group Interference}}{\underset{︸}{H_{k,u}\sum_{\begin{array}{c} j=1\\ j\neq k \end{array}}^{K}V_{j}F_{j}s_{j}}}+\underset{\mathrm{Noise}}{\underset{︸}{n_{k,u}}}, \end{array}$$

where $n_{k,u}$ is the complex additive white Gaussian noise with zero mean and variance equal to $\sigma_{k,u}^{2}$.

Assuming that the receivers are all low-cost terminals that perform analog beamforming only in decoding, the decoded signal denoted by ${\hat{s}}_{k,u}$ is given by:

(11) $${\hat{s}}_{k,u}=w_{k,u}^{H}H_{k,u}V_{k}f_{k,u}s_{k,u}+w_{k,u}^{H}{\tilde{n}}_{k,u},$$

where $w_{k,u}$ of length $N_{R}$ is the analog beamforming vector employed by the receiver with the power constraint of ${∥w_{k,u}∥}^{2}=1$ and

(12) $${\tilde{n}}_{k,u}=H_{k,u}V_{k}\sum_{\begin{array}{c} i=1\\ i\neq u \end{array}}^{M_{k}}f_{k,i}s_{k,i}+H_{k,u}\sum_{\begin{array}{c} j=1\\ j\neq k \end{array}}^{K}V_{j}F_{j}s_{j}+n_{k,u}.$$

Please note that the first term in Equation (11) stands for the desired signal while the second term the sum of inter- and intra-group interference as well as receiver thermal noise.

### 2.4. Group-By-Group Hybrid Precoding

For notational simplicity, we denote by $g_{k,u}^{(j)H}$ the effective analog beamforming gain vector observed by the *u*-th user in the *k*-th group from the *j*-th group for j,k=1,2,⋯,K.

(13) $$g_{k,u}^{(j)H}=w_{k,u}^{H}H_{k,u}V_{j}.$$

Let p be the transmitted power vector, where the power allocated to the *u*-th user in the *k*-th group is denoted by $p_{k,u}$ with

(14) $$\sum_{k=1}^{K}\sum_{u=1}^{M_{k}}p_{k,u}\leq N_{U}.$$

Then, the resulting channel capacity can be computed as

(15) $$R_{k,u}\;=\;\log_{2}\left(1+\frac{p_{k,u}{\left|g_{k,u}^{(k)H}f_{k,u}\right|}^{2}}{\zeta_{k,u}+\sigma_{k,u}^{2}}\right),$$

with

(16) $$\zeta_{k,u}=\sum_{\begin{array}{c} i=1\\ i\neq u \end{array}}^{M_{k}}p_{k,i}|g_{k,u}^{(k)H}f_{k,i}{|}^{2}+\;\sum_{\begin{array}{c} j=1\\ j\neq k \end{array}}^{K}\sum_{\begin{array}{c} t=1 \end{array}}^{M_{j}}p_{j,t}{\left|g_{k,u}^{(j)H}f_{j,t}\right|}^{2},$$

and $\sigma_{k,u}^{2}$ is the noise power.

Subsequently, the system sum-rate capacity can be computed as a function of W, V, F and p:

(17) $$R_{tot}\left(W,V,F,p\right)=\sum_{k=1}^{K}\sum_{u=1}^{M_{k}}R_{k,u}.$$

It is worth noting that the digital beamforming vectors can be designed to eliminate user interference for the conventional hybrid beamforming with sufficient RF chains, i.e.,

(18) $$\sum_{\begin{array}{c} i=1\\ i\neq u \end{array}}^{M_{k}}p_{k,i}|g_{k,u}^{(k)H}f_{k,i}{|}^{2}+\;\sum_{\begin{array}{c} j=1\\ j\neq k \end{array}}^{K}\sum_{\begin{array}{c} t=1 \end{array}}^{M_{j}}p_{j,t}{\left|g_{k,u}^{(j)H}f_{j,t}\right|}^{2}=0.$$

In contrast, it can only achieve interference-free asymptotically as $N_{T}$ grows a very large number since the proposed BD precoding scheme requires fewer RF chains, i.e., $N_{RF}<N_{U}$. Thus, the capacity of the proposed BD precoding scheme is constrained by the residual inter- and intra-group interference in the system. Given *K* groups, we can derive the optimal analog and block digital precoding matrices by

(19) $$\begin{array}{cc} P_{1}:\; & \underset{W,V,F,p}{\mathrm{maximize}}\;R_{tot}\left(W,V,F,p\right)\\ \mathrm{subject}\;\mathrm{to}\; & C_{1}:{\left|{\left[v_{k,u}\right]}_{i}\right|}^{2}=1/N_{T},i=1,2,\cdots ,N_{T};\\ \; & C_{2}:{\left|{\left[w_{k,u}\right]}_{j}\right|}^{2}=1/N_{R},j=1,2,\cdots ,N_{R};\\ \; & C_{3}:{∥V_{k}f_{k,u}∥}^{2}=1;\\ \; & C_{4}:V=\left[V_{1},V_{2},\cdots ,V_{K}\right];\\ \; & C_{5}:F=\mathrm{diag}\left(F_{1},F_{2},\cdots ,F_{K}\right);\\ \; & C_{6}:\max{\left\{M_{k}\right\}}_{k=1}^{K}\leq N_{RF};\\ \; & C_{7}:\sum_{k=1}^{K}\sum_{u=1}^{M_{k}}p_{k,u}\leq N_{U};\\ \; & C_{8}:R_{k,u}\geq \lambda_{k,u}, \end{array}$$

where k=1,2,⋯,K and $u=1,2,\cdots ,M_{k}$ in $C_{1}$, $C_{2}$ and $C_{3}$.

In problem $P_{1}$, $C_{1}$ and $C_{2}$ confine the analog beamforming vectors to the phase-only structure in transmitter and receiver while $C_{3}$ ensures that each precoded signal is of unit power. Furthermore, $C_{4}$ and $C_{5}$ define the analog and digital precoder, respectively. $C_{6}$ constrains the maximum number of data-streams in each group to be within the number of RF chains. Finally, $C_{7}$ defines the downlink transmitted power constraint while $C_{8}$ guarantees the minimal data rate $\lambda_{k,u}$ for each user.

The problem $P_{1}$ is challenging due to its non-convex and combinatorial nature. Thus, it is analytically intractable to derive a closed-form optimal solution. Instead, we consider a two-stage suboptimal solution: In the first stage, we focus on the analog and digital precoder design while assuming uniform power allocation; After fixing the analog and digital precoders, we derive the QoS-aware optimal power allocation in the second stage.

## 3. Proposed Block Hybrid Beamforming for RF Chains Reduction

In this section, we will first ignore the constraints $C_{7}$ and $C_{8}$ in $P_{1}$ by uniformly allocating the power to each user while assuming that the user grouping is given.

### 3.1. Analog Beamforming Design

We begin with the analog beamforming design for both transmitter and receiver. It is well known that distinct array response vectors are asymptotically orthogonal as the number of antennas in an antenna array goes to infinity [1], i.e.,

(20) $$\lim_{N\to +\infty }a_{T}^{H}\left(\phi_{k,u}^{t},\theta_{k,u}^{t}\right)\cdot a_{T}\left(\phi_{\ell ,v}^{t},\theta_{\ell ,v}^{t}\right)=\delta \left(k-\ell \right)\delta \left(u-v\right).$$

However, since the antenna number is finite in practice, the residual interference must be considered in the analog precoding design. Recalling the channel model presented in Equation (8), we can asymptotically orthogonalize the transmitted signals by optimizing the design of $w_{k,u}$ and $v_{k,u}$:

(21) $$\begin{array}{cc} \left\{w_{k,u}^{*},v_{k,u}^{*}\right\} & =\underset{{\tilde{w}}_{k,u},{\tilde{v}}_{k,u}}{\arg\;\max}\sum_{k=1}^{K}\sum_{u=1}^{M_{k}}\log_{2}\left(1+\mathrm{SINR}\left({\tilde{w}}_{k,u},{\tilde{v}}_{k,u}\right)\right)\\ \mathrm{subject}\;\mathrm{to} & \;{\tilde{v}}_{k,u}\in A_{k,u}^{T};\\ \; & \;{\tilde{w}}_{k,u}\in A_{k,u}^{R};\\ \; & \;\max{\left\{M_{k}\right\}}_{k=1}^{K}<N_{RF}, \end{array}$$

where

(22a) $$\begin{array}{c} A_{k,u}^{T}=\left[a_{T}\left(\phi_{k,u,1}^{t},\theta_{k,u,1}^{t}\right),\cdots ,a_{T}\left(\phi_{k,u,L_{k,u}}^{t},\theta_{k,u,L_{k,u}}^{t}\right)\right], \end{array}$$

(22b) $$\begin{array}{c} A_{k,u}^{R}=\left[a_{R}\left(\phi_{k,u,1}^{r},\theta_{k,u,1}^{r}\right),\cdots ,a_{R}\left(\phi_{k,u,L_{k,u}}^{r},\theta_{k,u,L_{k,u}}^{r}\right)\right]. \end{array}$$

Furthermore, ${\mathrm{SINR}}_{k,u}$ is given by

(23) $$\mathrm{SINR}\left({\tilde{w}}_{k,u},{\tilde{v}}_{k,u}\right)=\frac{|{\tilde{w}}_{k,u}^{H}H_{k,u}{\tilde{v}}_{k,u}{|}^{2}}{\sum_{j=1,j\neq k}^{N_{U}}{∥{\tilde{w}}_{k,u}^{H}H_{k,u}{\tilde{V}}_{j}∥}^{2}+\sum_{t\neq u}^{M_{k}}{\left|{\tilde{w}}_{k,u}^{H}H_{k,u}{\tilde{v}}_{k,t}\right|}^{2}+\frac{1}{\gamma }},$$

with $\gamma =\frac{1}{\sigma_{k,u}^{2}}$. The optimal analog beamforming precoder can be straightforwardly found by exhaustively searching in the feasible sets of $A_{k,u}^{T}$ and $A_{k,u}^{R}$.

### 3.2. Digital Precoder Design

In this section, two digital precoding schemes are proposed to maximize the system sum-rate by minimizing the intra-group interference.

#### 3.2.1. Block Zero-Forcing (Bzf) Scheme

In contrast to the conventional ZF hybrid beamforming scheme [28] that requires $N_{U}\leq N_{RF}$, zero-forcing digital precoding scheme is first proposed to transmit data-streams group by group. More specifically, the digital precoder for each block is designed as the inverse of the effective channel of the block:

(24) $$F_{k}^{\mathrm{BZF}}=G_{k}^{H}{\left(G_{k}G_{k}^{H}\right)}^{-1},$$

with $N_{RF}\geq M_{k}$, where $G_{k}={\left[g_{k,1}^{\left(k\right)},g_{k,2}^{\left(k\right)},\cdots ,g_{k,M_{k}}^{\left(k\right)}\right]}^{H}$.

To satisfy the constraint $C_{3}$ in $P_{1}$, power normalization is performed on each $f_{k,u}$ derived from $F_{k}^{\mathrm{BZF}}=\left[f_{k,1}^{\mathrm{BZF}},f_{k,2}^{\mathrm{BZF}},\cdots ,f_{k,M_{k}}^{\mathrm{BZF}}\right]$ as

(25) $${\bar{f}}_{k,u}^{\mathrm{BZF}}=\frac{f_{k,u}^{\mathrm{BZF}}}{∥V_{k}\cdot f_{k,u}^{\mathrm{BZF}}∥}.$$

Subsequently, this scheme is referred to as the block zero-forcing (BZF) scheme. It is worth noting that BZF degenerates to [28] if K=1, i.e., all users are grouped into one independent group. On the other hand, BZF becomes the analog-only BDMA if $K=N_{U}$, i.e., each user forms one group and only analog beamforming is performed.

#### 3.2.2. Block SLNR Maximization (BSM) Scheme

Instead of the received interference elimination, we can alternatively devise the digital precoder to suppress the interference leakage by maximizing SLNR [29]. More specifically, we denote by $P_{k,u}^{\mathrm{Desired}}$ the desired signal power transmitted to the *u*-th user in the *k*-th group

(26) $$P_{k,u}^{\mathrm{Desired}}=\gamma {\left|g_{k,u}^{(k)H}f_{k,u}^{\mathrm{BSM}}\right|}^{2}.$$

Furthermore, if we define leakage signal as the transmitted signal that is intended to a specific user but leaked to other users, the leakage signal power from the *u*-th user in the *k*-th group can be given as

(27) $$\begin{array}{cc} P_{k,u}^{\mathrm{Leakage}}= & \gamma \left(\sum_{\begin{array}{c} j=1\\ j\neq k \end{array}}^{K}\sum_{i=1}^{M_{j}}{\left|g_{j,i}^{(k)H}f_{k,u}^{\mathrm{BSM}}\right|}^{2}+\sum_{\begin{array}{c} t=1\\ t\neq u \end{array}}^{M_{k}}{\left|g_{k,t}^{(k)H}f_{k,u}^{\mathrm{BSM}}\right|}^{2}\right). \end{array}$$

Finally, the SLNR for the *u*-th user in the *k*-th group can be formulated as

(28) $$\Gamma_{k,u}=\frac{{\left|g_{k,u}^{(k)H}f_{k,u}^{\mathrm{BSM}}\right|}^{2}}{\sum_{\begin{array}{c} j=1\\ j\neq k \end{array}}^{K}\sum_{i=1}^{M_{j}}{\left|g_{j,i}^{(k)H}f_{k,u}^{\mathrm{BSM}}\right|}^{2}+\sum_{\begin{array}{c} t=1\\ t\neq u \end{array}}^{M_{k}}{\left|g_{k,t}^{(k)H}f_{k,u}^{\mathrm{BSM}}\right|}^{2}+\frac{1}{\gamma }}.$$

Denoted by $f_{k,u}^{\mathrm{BSM}}$ the optimal digital precoder maximizing SLNR, it has been shown that $f_{k,u}^{\mathrm{BSM}}$ turns out to be the eigenvector associated with the largest eigenvalue of the following matrix [29]:

(29) $$R_{k,u}^{\mathrm{Leakage}}={\left(\frac{1}{\gamma }I_{N_{RF}}+Q_{k,u}\right)}^{-1}g_{k,u}^{\left(k\right)}g_{k,u}^{(k)H},$$

where $Q_{k,u}$ is the leakage covariance matrix related to the *u*-th user in the *k*-th group and given as:

(30) $$Q_{k,u}=\sum_{\begin{array}{c} j=1\\ j\neq k \end{array}}^{K}\sum_{i=1}^{M_{j}}g_{j,i}^{\left(k\right)}g_{j,i}^{(k)H}+\sum_{\begin{array}{c} t=1\\ t\neq u \end{array}}^{M_{k}}g_{k,t}^{\left(k\right)}g_{k,t}^{(k)H}.$$

In contrast to the conventional hybrid precoding algorithms with complexity $O(N_{U}^{3})$, the proposed group precoding schemes can reduce the complexity to $O(N_{RF}^{3})$.

From the above derivation, it is apparent that the user grouping algorithm plays an important role on the amount of inter-group interference, and subsequently the system performance. Thus, a heuristic algorithm for user grouping is investigated in the next section.

## 4. User Grouping Algorithm

Since the intra-group interference is eliminated by the digital precoding, we will focus on using the user grouping to maximize the intra-group interference while minimizing the inter-group interference. More specifically, we propose to group $N_{U}$ users into *K* groups with minimal inter-group interference. Since the total number of possible group combinations is large, a greedy grouping algorithm is proposed in Algorithm 1.

**Algorithm 1** Greedy User Grouping Algorithm
**Input:**
X: the universal group and user index set;$a_{T}\left(\phi_{x}^{t},\theta_{x}^{t}\right)$: Array response vector of index *x*;$I_{k}=\emptyset$: the user index set for the *k*-th group;k=1: group index;Initialize $I_{1}\leftarrow x^{*}$ with $x^{*}$ being the user index of the largest channel gain and $X\setminus x^{*}$;
**Procedures:**
  1:**while**X is not empty **do**  2:*Stage 1*:  3:   Solve the optimal analog precoder by Equation (21)  4:   Let A be the analog precoders of grouped users  5:   **for** *x* in X **do**  6:      Compute $S\left(x\right)={∥a_{T}^{H}\left(\phi_{x}^{t},\theta_{x}^{t}\right)\cdot A∥}^{2}$  7:   **end for**  8:   Find the user index $x^{*}$ with *maximum*S(x)  9:   Update $A\leftarrow x^{*}$, $I_{k}\leftarrow x^{*}$ and $X\setminus x^{*}$ 10:*Stage 2*: 11:   **if** $|I_{k}|=N_{RF}$ **then** 12:      Update k←k+1 13:      **for** *x* in X **do** 14:         Compute $S\left(x\right)=∥a_{T}^{H}\left(\phi_{x}^{t},\theta_{x}^{t}\right){\cdot A∥}^{2}$ 15:      **end for** 16:      Find the user index $x^{*}$ with *minimum*S(x) 17:      Update $A\leftarrow x^{*}$, $I_{k}\leftarrow x^{*}$ and $X\setminus x^{*}$ 18:   **end if** 19:
**end while**



In this algorithm, for k=1, we first group users who cause most interference to each other into Group 1 detailed in Stage 1. This selection is motivated by the observation that most interference can be eliminated by the digital precoder applied among each group. When the size of Group 1 reaches the number of RF chains, the user whose array response vector is most orthogonal to Group 1 is selected as the first member of Group 2 as shown in Stage 2, which is designed to minimize the inter-group interference. This process repeats until all users are assigned to different groups.

It is worth noting that the grouping problem is NP-hard. The greedy algorithm is proposed to find a suboptimal partition with complexity $O(N_{U}^{2})$.

## 5. Performance Analysis

We first investigate the capacity for the conventional analog-only BDMA scheme.

(31) $$\begin{array}{cc} E[R_{k,u}]= & E\left[\log_{2}\left(1+\mathrm{SINR}\right)\right], \end{array}$$

(32) $$\begin{array}{cc} \;\;\;= & E\left[\log_{2}\left(1+\frac{|w_{k,u}^{*H}H_{k,u}v_{k,u}^{*}{|}^{2}}{1/\gamma +I_{k,u}}\right)\right], \end{array}$$

where $I_{k,u}$ is the received interference represented as

(33) $$\begin{array}{c} I_{k,u}=\sum_{j\neq k}^{K}∥w_{k,u}^{*H}H_{k,u}V_{j}{∥}^{2}+\sum_{t\neq u}^{M_{k}}{\left|w_{k,u}^{*H}H_{k,u}v_{k,t}\right|}^{2}. \end{array}$$

**Proposition** **1.** If the optimal analog beamformers are designed as $w_{k,u}^{*}=a_{R}\left(\phi_{k,u,l^{*}}^{r},\theta_{k,u,l^{*}}^{r}\right)$ and $v_{k,u}^{*H}=a_{T}^{H}\left(\phi_{k,u,l^{*}}^{t},\theta_{k,u,l^{*}}^{t}\right)$, respectively, the following approximation holds:

(34) $$\begin{array}{c} |w_{k,u}^{*H}H_{k,u}v_{j,i}{|}^{2}\approx {\left|D_{k,u}\alpha_{k,u,l^{*}}v_{k,u}^{*H}v_{j,i}\right|}^{2}. \end{array}$$

The proof is given in Appendix A and Appendix B.

From Proposition 1, Equation (31) can be rewritten as

(35) $$E\left[R_{k,u}\right]=E\left[\log_{2}\left(1+\frac{Z}{1/\gamma +Y}\right)\right],$$

where

(36) $$\begin{array}{cc} Z & =|w_{k,u}^{*H}H_{k,u}v_{k,u}^{*}{|}^{2}, \end{array}$$

(37) $$\begin{array}{cc} \; & \approx D_{k,u}^{2}\alpha_{k,u,l^{*}}^{2}, \end{array}$$

and

(38) $$\begin{array}{cc} Y & \approx D_{k,u}^{2}\alpha_{k,u,l^{*}}^{2}\left(\sum_{j\neq k}^{K}{∥v_{k,u}^{*H}V_{j}∥}^{2}+\sum_{t\neq u}^{M_{k}}{\left|v_{k,u}^{*H}v_{k,t}\right|}^{2}\right),\\ \; & \approx Z\left(\sum_{j\neq k}^{K}{∥v_{k,u}^{*H}V_{j}∥}^{2}+\sum_{t\neq u}^{M_{k}}{\left|v_{k,u}^{*H}v_{k,t}\right|}^{2}\right). \end{array}$$

Capitalizing on the Extreme Value Theory [30,31], we can derive the cumulative distribution function (CDF) of *Z* as

(39) $$F_{Z}\left(z\right)={\left(1-e^{-\frac{z}{C}}\right)}^{L_{k,u}},$$

where $C=2N_{T}N_{R}/L_{k,u}$. The detailed derivation is shown in Appendix C.

The residual interference of distinct beams is negligible as compared to the desired signal. Thus, *Y* can be upper bounded by

(40) $$\begin{array}{cc} Y\leq & Z\cdot E\left[\sum_{j\neq k}^{K}{\left|v_{k,u}^{*H}V_{j}\right|}^{2}+\sum_{t\neq u}^{M_{k}}{\left|v_{k,u}^{*H}v_{k,t}\right|}^{2}\right], \end{array}$$

(41) $$\begin{array}{cc} \approx & Z\left(N_{U}-1\right)\cdot E\left[|v_{k,u}^{*H}v_{j,i}{|}^{2}\right], \end{array}$$

(42) $$\begin{array}{cc} = & Z(N_{U}-1)T, \end{array}$$

where 0≤T≤1 with *T* being the expected residual interference power between distinct beams. In our proposed system, the beams will be selected and grouped to reduce the residual interference. Clearly, T=0 if the number of antennas goes to infinity or the steering vectors of different users are strictly orthogonal. In contrast, T=1 if different users have same AoDs. The value of *T* can be numerically derived.

Finally, the CDF of the SINR lower bound can be given by

(43) $$F_{X}\left(x\right)={\left(1-e^{-\frac{x}{C\gamma (1-T\left(N_{U}-1\right)x)}}\right)}^{L_{k,u}}.$$

The detailed derivation can be found in Appendix D.

Using the CDF above, the lower and upper bounds of the sum-rate capacity can be derived as

(44) $$\int_{0}^{\infty }\log_{2}\left(1+x\right)dF_{X}\left(x\right)\leq E\left[R_{k,u}\right]\leq \int_{0}^{\infty }\log_{2}\left(1+z\right)dF_{Z}\left(z\right).$$

It is analytically intractable to obtain a closed-form solution to Equation (44). We will show the numerical results in simulation section.

Please note that the upper bound is achieved if the interference from other users can be eliminated. Furthermore, since the number of transmitter antennas is finite in practice, the analog beamforming vectors shown in Equations (21) and (23) inevitably incur residual inter-user interference. Therefore, digital precoders are required to further suppress the residual interference.

## 6. Proposed QoS-Aware Power Allocation Algorithm Based on D.C. Programming

For given analog and digital precoders, we investigate the QoS-aware power allocation p in $P_{1}$ by using the D.C. programming technique in this section.

We begin with reformulating $P_{1}$ as

(45) $$\begin{array}{cc} \; & \underset{p}{\mathrm{maximize}}\;\sum_{k=1}^{K}\sum_{u=1}^{M_{k}}R_{k,u}\left(p\right)\\ \mathrm{subject}\;\mathrm{to}\; & C_{1}:\sum_{k=1}^{K}\sum_{u=1}^{M_{k}}p_{k,u}\leq P;\\ \; & C_{2}:R_{k,u}\geq \lambda_{k,u}, \end{array}$$

Following the procedures in [32], the problem above can be cast as a D.C. programming problem:

(46) $$\;\underset{p}{\mathrm{maximize}}\;f\left(p\right)-g\left(p\right)$$

where

$$\begin{array}{cc} f(p)= & \sum_{k=1}^{K}\sum_{u=1}^{M_{k}}\log_{2}\left(\sum_{\begin{array}{c} k=1 \end{array}}^{K}\sum_{\begin{array}{c} u=1 \end{array}}^{M_{k}}p_{k,u}{\left|g_{k,u}^{(k)H}f_{k,u}\right|}^{2}+\sigma_{k,u}^{2}\right),\\ g(p)= & \sum_{k=1}^{K}\sum_{u=1}^{M_{k}}\log_{2}\left(\sum_{\begin{array}{c} j=1\\ j\neq k \end{array}}^{K}\sum_{\begin{array}{c} t=1 \end{array}}^{M_{j}}p_{j,t}{\left|g_{k,u}^{(j)H}f_{j,t}\right|}^{2}\;+\sigma_{k,u}^{2}\right). \end{array}$$

For given analog and digital precoders, both f(p) and g(p) are concave in p, i.e., Equation (46) is a D.C. function. Starting from a feasible $p^{\left(0\right)}$, the optimal $p^{\left(n+1\right)}$ at the *n*-th iteration is generated as the optimal solution of a convex problem:

(47) $$\max_{p}f\left(p\right)-g\left(p^{n}\right)-\langle ▽g\left(p^{\left(n\right)}\right),p-p^{\left(n\right)}\rangle ,$$

which can be efficiently solved by any existing convex programming software, such as CVX [33]. The computational complexity of Equation (47) is $O(N_{RF}^{3})$ in each iteration [32].

As $g(p^{n})$ is concave, its gradient $▽g(p^{\left(n\right)})$ is also super-gradient:

(48) $$\begin{array}{cc} \; & f\left(p^{\left(n+1\right)}\right)-g\left(p^{\left(n+1\right)}\right)\geq \\ \; & f\left(p^{\left(n+1\right)}\right)-\left[g\left(p^{\left(n\right)}\right)+\langle ▽g\left(p^{\left(n\right)}\right),p^{\left(n+1\right)}-p^{\left(n\right)}\rangle \right]. \end{array}$$

The proof is given in Appendix E.

Finally, since $p^{\left(n+1\right)}$ is the solution to Equation (47), it follows that

(49a)f(p(n+1))−g(p(n))−〈▽g(p(n)),p(n+1)−p(n)〉,(49b)≥f(p(n))−g(p(n))−〈▽g(p(n)),p(n)−p(n)〉,(49c)=f(p(n))−g(p(n)).

Therefore, the (n+1)-th solution is always better than the previous one. The iterative process terminates after $|f\left(p^{\left(n+1\right)}\right)-g\left(p^{\left(n+1\right)}\right)-\left(f\left(p^{\left(n\right)}\right)-g\left(p^{\left(n\right)}\right)\right)|\leq \epsilon$ is achieved with a pre-defined threshold ϵ>0.

## 7. Simulation Results

In this section, we will use computer simulation to evaluate the sum-rate performance of the proposed block diagonal digital precoding schemes. Unless specified otherwise, we consider a transmitter equipped with a 12×12 UPA (i.e., $N_{T}=144$) and $N_{U}=16$ users each equipped with an 8×8 UPA (i.e., $N_{R}=64$). The number of paths is set to $L_{k,u}=4$ and the additive Gaussian noise power $\sigma_{k,u}^{2}=$−30 dBm for each user. We consider the azimuth AoA/AoD’s uniformly distributed over [0,2π] while the elevation AoA/AoD’s uniformly distributed over [−π/2,π/2], respectively. For each computer experiment, we compute the average over 100 realizations.

**Figure 2:**
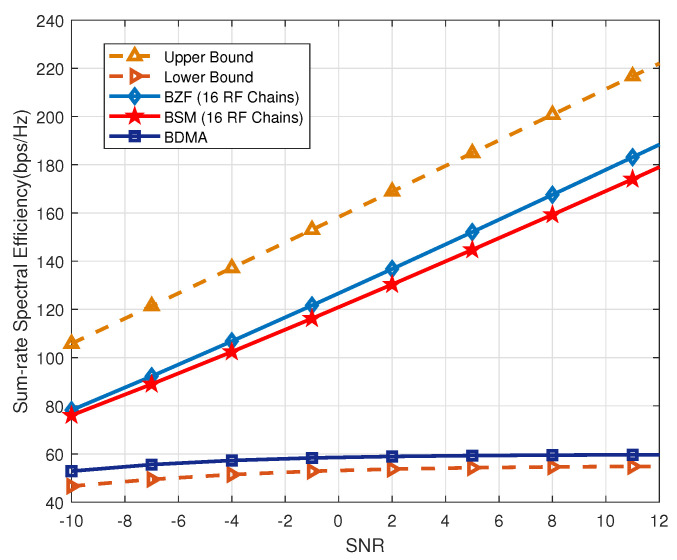
Performance with K=1 (no grouping) over multipath channels.

In Figure 2, we first set K=1, i.e., no grouping. As a result, 16 RF chains are required to support 16 data-streams. As shown in Figure 2, BZF slightly outperforms BSM as it can eliminate more multi-user interference even in multipath environment. It is observed that even in the high SNR regime, BDMA suffers from inter-beam interference and has poor performance.

**Figure 3:**
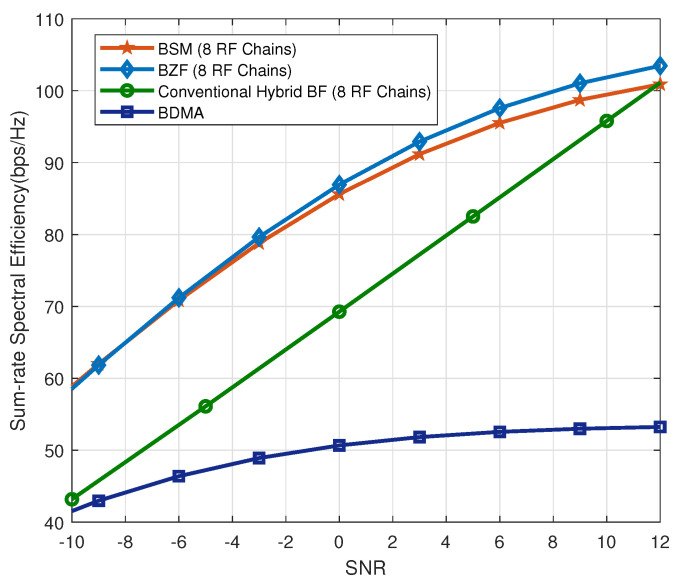
Performance with K=2 groups over multipath channels with $N_{u}=16$ users and 8 RF chains.

Next, we evaluate the two proposed BD precoding schemes with K=2 groups and 8 RF chains. The 16 users are grouped into K=2 groups. The curves labeled as “BZF” and “BSM” stands for the proposed BD precoding schemes where only 8 RF chains are used to transmit 16 data-streams. It is observed that BZF and BSM have comparable performance. Furthermore, the curve labeled as “Conventional Hybrid BF (8 RF Chains)” is the sum-rate for the conventional hybrid beamforming system with 8 RF chains serving 8 users. Finally, BDMA is the analog-only precoding system that has the worst performance. Inspection of Figure 3 reveals that the proposed BZF and BSM have much better sum-rate performance than the conventional hybrid precoding algorithm over the SNR range [−10,10] dB. When the SNR is larger than 12 dB, the system becomes interference-limited. Thus, the performance of BZF and BSM tends to saturate beyond this point.

**Figure 4:**
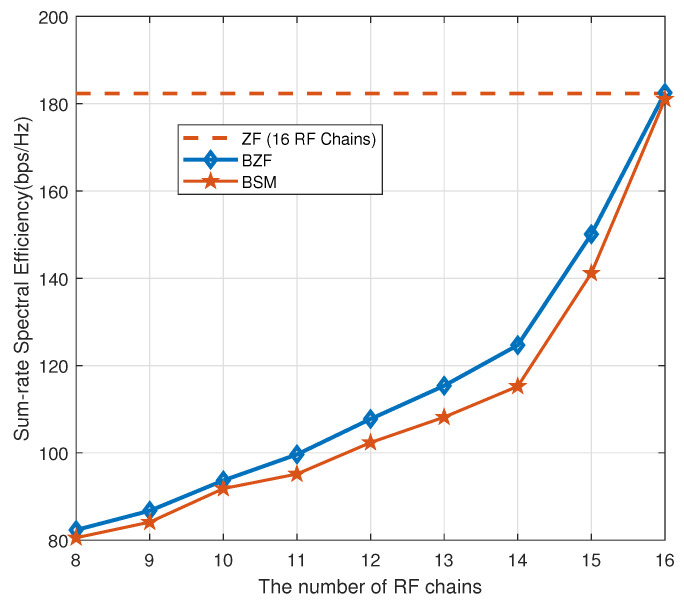
Sum-rate capacity improvement as a function of the number of RF chains.

In Figure 4, we investigate the sum-rate capacity improvement as a function of the number of RF chains while fixing the SNR at 5 dB. The upper bound is the conventional ZF precoding system with 16 RF chains for 16 users. Interestingly, the performance improvement generated by an additional RF chain increases only marginally as the number of RF chains grows from eight to 14.

**Figure 5:**
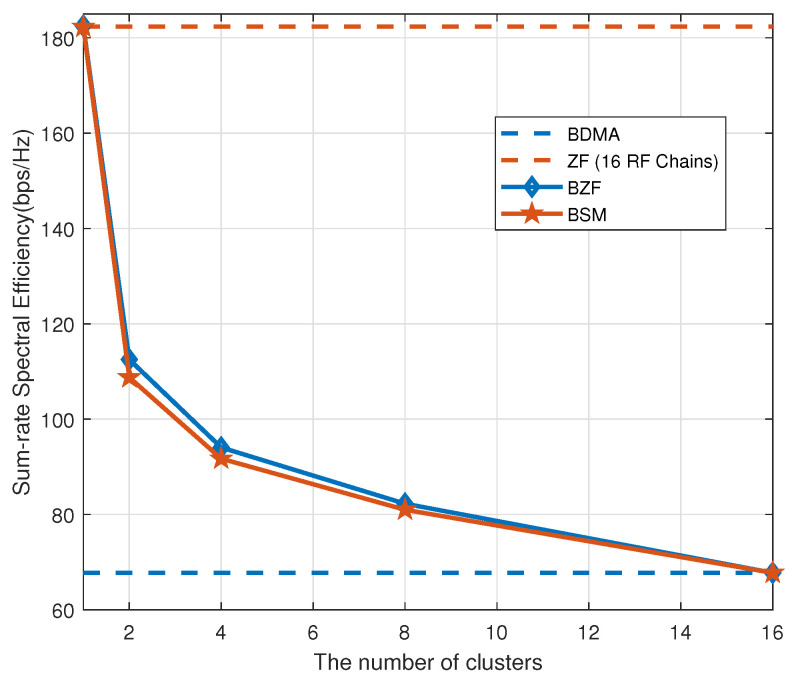
Sum-rate capacity as a function of the number of groups.

Next, we vary the number of groups while fixing the total number of users at $N_{u}=16$ and SNR =5 dB. Figure 5 shows that BZF and BSM are lower bounded by BDMA and upper bounded by the conventional ZF system with 16 RF chains. When K=1, the system degenerates back to the conventional ZF system with $N_{RF}=M_{1}=16$. On the other hand, if K=16, the system becomes the conventional BDMA.

**Figure 6:**
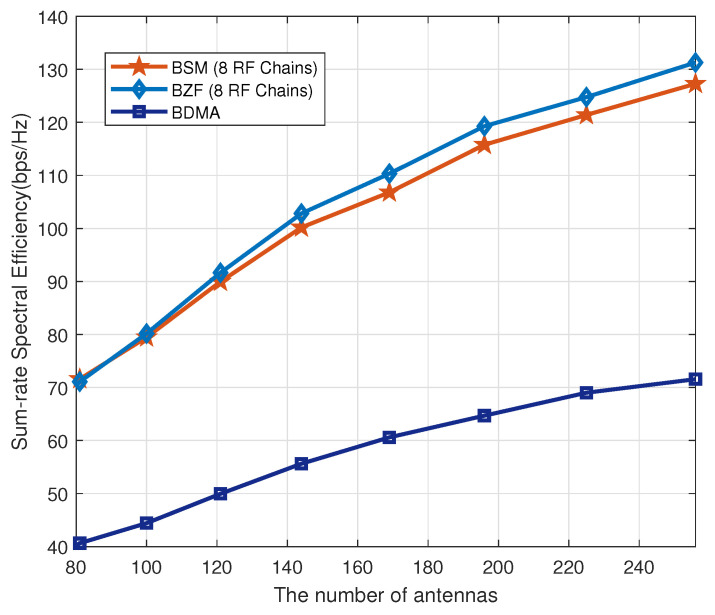
Sum-rate capacity as a function of the number of transmit antennas.

We then investigate the sum-rate performance as the number of transmit antennas increases. Figure 6 shows that the capacity of BZF and BSM has been significantly increased as the number of transmit antennas increases. This is because that the inter-group interference is asymptotically removed as indicated in Equation (20).

**Figure 7:**
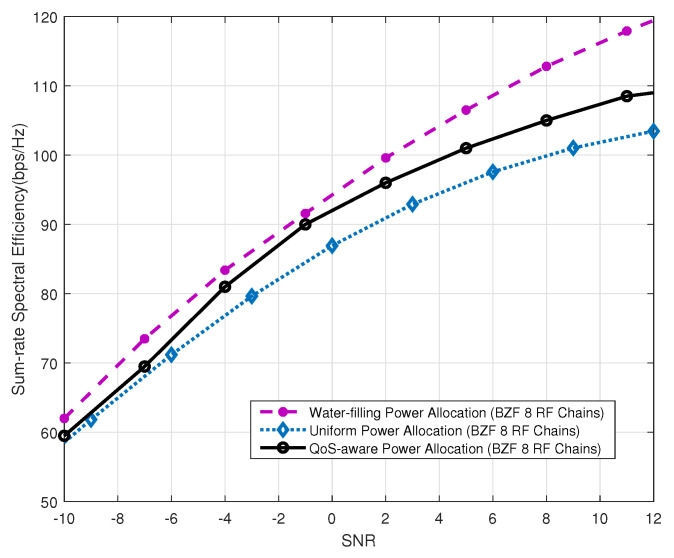
Performance achieved by the QoS-aware power allocation algorithm with K=2.

**Figure 8:**
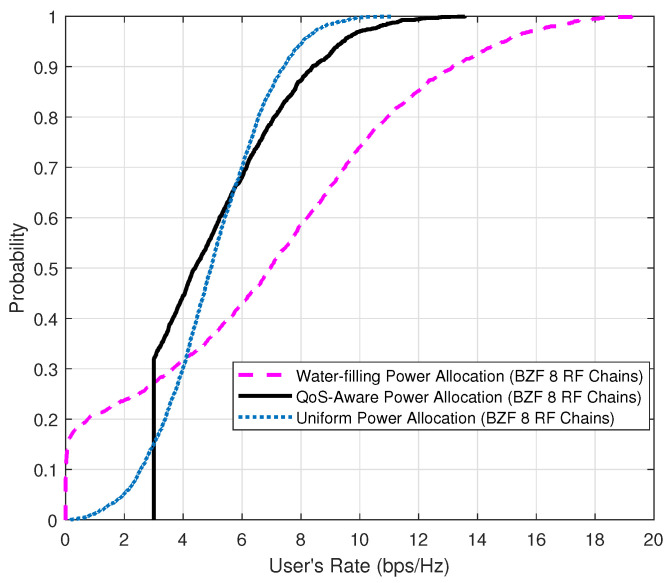
The CDF of user’s rate comparison for QoS-ware power allocation schemes.

Finally, we evaluate the performance of the power allocation generated with the D.C. programming technique. We assume that the minimum QoS threshold for each user is set to 3 bps/Hz. Figure 7 and Figure 8 show the performance achieved by our proposed QoS-aware power allocation algorithm. The curve labeled as “Water-filling Power Allocation” is obtained by allocating user power via the water-filling algorithm without taking into account the QoS requirement. The curve labeled as “QoS-Aware Power Allocation” shows the performance of the proposed power allocation algorithm. Compared to the curve labeled as “Uniform Power Allocation”, the proposed algorithm has demonstrated significant advantages in terms of the sum-rate capacity. Furthermore, Figure 8 depicts the CDF of the user data rate. It is evident that all users served by the QoS-aware power allocation satisfy the minimum QoS requirement (i.e., 3 bps/Hz). In contrast, the water-filling-based power algorithm suffers from an outage rate of about 20% where outage is defined as the user data rate being below the minimum required data rate.

## 8. Conclusions

In this paper, we have developed block diagonal hybrid precoding schemes with optimized power allocation for mmWave massive MIMO systems by jointly performing hybrid analog-digital precoding and user-beam grouping. The proposed system requires fewer RF chains as compared to the conventional hybrid precoding systems by digitally precoding data-streams group by group. Although the intra-group interference is eliminated by the digital precoding, a greedy grouping algorithm has been derived to minimize the inter-group interference by carefully grouping users with orthogonal beams to different groups. Furthermore, two digital precoding schemes have been proposed to suppress the intra-group interference based on SINR and SLNR, respectively. In addition, the upper and lower bounds of the system sum-rate capacity have been derived based on the multipath channel model. Finally, QoS-aware power allocation has been proposed by using the D.C. programming technique. Simulation results have demonstrated the good performance of the proposed grouped BDMA block diagonal hybrid precoding system.

## Figures and Tables

**Figure A1:**
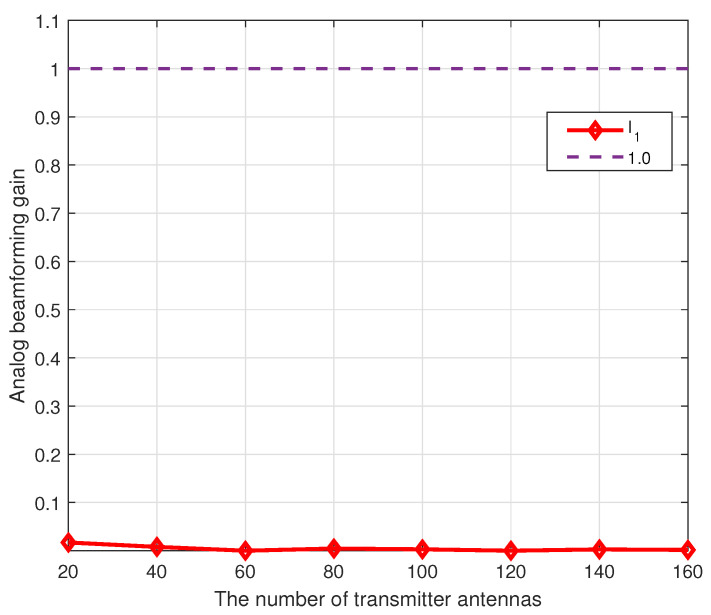
Desired signal from different paths.

**Figure A2:**
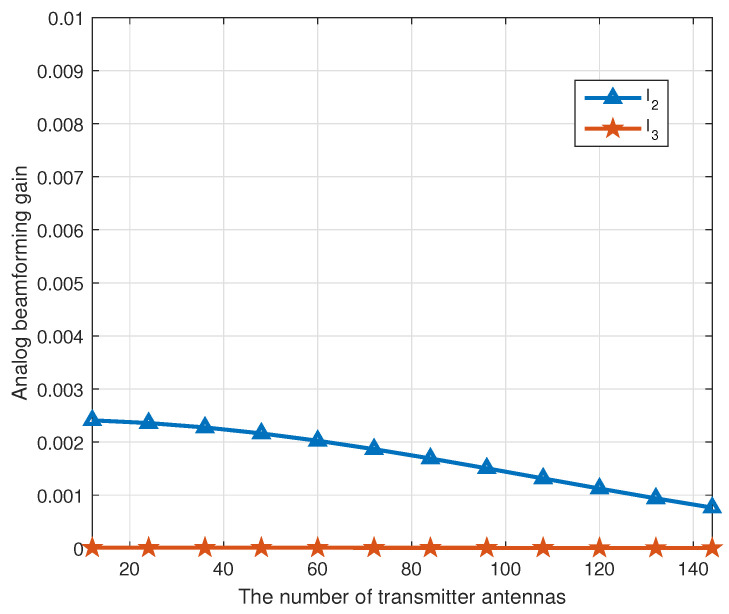
Other users’ interference from different paths.

**Figure A3:**
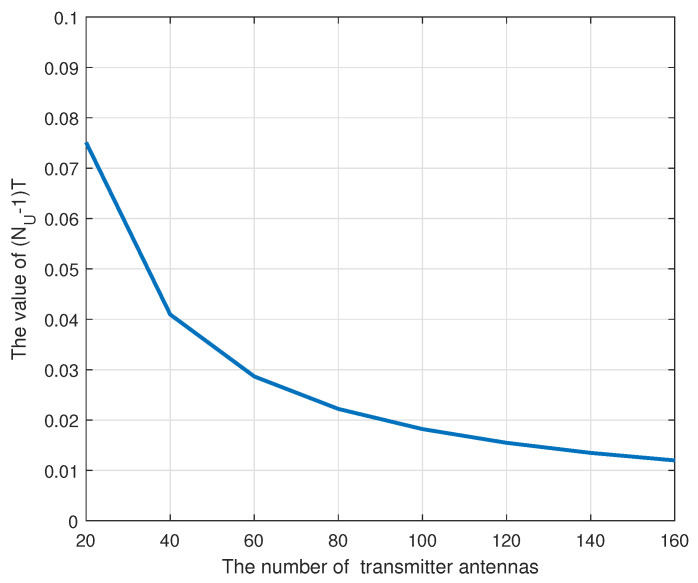
The value of $(N_{U}-1)T$ for different number of transmitter antennas.

## Appendix A. Proof of Proposition 1

**Proof.** First, if $v_{j,i}=v_{k,u}^{*}=a_{T}^{H}\left(\phi_{k,u,l^{*}}^{t},\theta_{k,u,l^{*}}^{t}\right)$, we show that the signal power received from other paths is much smaller than that from the optimal path,

(A1) $$\begin{array}{cc} \; & |w_{k,u}^{*H}H_{k,u}v_{j,i}{|}^{2}\\ = & {\left|D_{k,u}w_{k,u}^{*H}\sum_{l=1}^{L_{k,u}}\alpha_{k,u,l}a_{R}\left(\phi_{k,u,l}^{r},\theta_{k,u,l}^{r}\right)a_{T}^{H}\left(\phi_{k,u,l}^{t},\theta_{k,u,l}^{t}\right)v_{k,u}^{*}\right|}^{2},\\ = & D_{k,u}^{2}\alpha_{k,u,l^{*}}^{2}{\left|v_{k,u}^{*H}v_{k,u}^{*}+I_{1}\right|}^{2}, \end{array}$$

where

(A2) $$\begin{array}{cc} \; & v_{k,u}^{*H}v_{k,u}^{*}=1.0,\\ \; & I_{1}=\sum_{l\neq l^{*}}^{L_{k,u}}\frac{\alpha_{k,u,l}}{\alpha_{k,u,l^{*}}}w_{k,u}^{*H}a_{R}\left(\phi_{k,u,l}^{r},\theta_{k,u,l}^{r}\right)a_{T}^{H}\left(\phi_{k,u,l}^{t},\theta_{k,u,l}^{t}\right)v_{k,u}^{*}. \end{array}$$

It can be easily shown that $I_{1}\ll 1.0$, which means the desired signal in Equation (A1) can be well approximated by $|D_{k,u}\alpha_{k,u,l^{*}}v_{k,u}^{*H}v_{k,u}^{*}{|}^{2}$. In other words, the vast majority of desired signal is received from the selected optimal path. Similarly, for $v_{j,i}\neq a_{T}^{H}\left(\phi_{k,u,l^{*}}^{t},\theta_{k,u,l^{*}}^{t}\right)$, the interference from another user is given by

(A3) $$\begin{array}{cc} \; & |w_{k,u}^{*H}H_{k,u}v_{j,i}{|}^{2}\\ = & {\left|D_{k,u}w_{k,u}^{*H}\sum_{l=1}^{L_{k,u}}\alpha_{k,u,l}a_{R}\left(\phi_{k,u,l}^{r},\theta_{k,u,l}^{r}\right)a_{T}^{H}\left(\phi_{k,u,l}^{t},\theta_{k,u,l}^{t}\right)v_{j,i}\right|}^{2},\\ = & D_{k,u}^{2}{\left|I_{2}+I_{3}\right|}^{2}, \end{array}$$

where

$$\begin{array}{cc} I_{2}= & \alpha_{k,u,l}a_{T}^{H}\left(\phi_{k,u,l}^{t},\theta_{k,u,l}^{t}\right)v_{j,i},\\ I_{3}= & \sum_{l\neq l^{*}}^{L_{k,u}}\alpha_{k,u,l}w_{k,u}^{*H}a_{R}\left(\phi_{k,u,l}^{r},\theta_{k,u,l}^{r}\right)a_{T}^{H}\left(\phi_{k,u,l}^{t},\theta_{k,u,l}^{t}\right)v_{j,i}. \end{array}$$

Since analog beamformers are distinct for different receivers, we have $I_{3}\ll I_{2}$ by Equation (20). Finally, it is straightforward to derive

(A4) $$\begin{array}{cc} \; & |w_{k,u}^{*H}H_{k,u}v_{j,i}{|}^{2}\\ = & |w_{k,u}^{*H}D_{k,u}\sum_{l=1}^{L_{k,u}}\alpha_{k,u,l}a_{R}\left(\phi_{k,u,l}^{r},\theta_{k,u,l}^{r}\right)a_{T}^{H}\left(\phi_{k,u,l}^{t},\theta_{k,u,l}^{t}\right)v_{j,i}{|}^{2},\\ \approx & |w_{k,u}^{*H}D_{k,u}\alpha_{k,u,l^{*}}a_{R}\left(\phi_{k,u,l^{*}}^{r},\theta_{k,u,l^{*}}^{r}\right)a_{T}^{H}\left(\phi_{k,u,l^{*}}^{t},\theta_{k,u,l^{*}}^{t}\right)v_{j,i}{|}^{2},\\ = & |D_{k,u}\alpha_{k,u,l^{*}}a_{T}^{H}\left(\phi_{k,u,l^{*}}^{t},\theta_{k,u,l^{*}}^{t}\right)v_{j,i}{|}^{2},\\ = & |D_{k,u}\alpha_{k,u,l^{*}}v_{k,u}^{*H}v_{j,i}{|}^{2}. \end{array}$$

A numerical example is given in Appendix B. □

## Appendix B. A Numerical Example for Proposition 1

A numerical example is given below. We set $L_{k,u}=2$ with $\phi_{k,u,1}=\phi_{k,u,1}=\pi /2$, $\theta_{k,u,1}=\pi /3$ and $\theta_{k,u,2}=\pi /5$. Recalling the given UPA array response vector, the number of antennas in transmitter is $N_{T}=144$ and $N_{R}=64$ in receiver. The optimal analog precoder in transmitter and receiver is given by $v_{k,u}^{*}=a_{T}\left(\phi_{k,u,1},\theta_{k,u,1}\right)$ and $w_{k,u}^{*}=a_{R}\left(\phi_{k,u,1},\theta_{k,u,1}\right)$, respectively. Furthermore, we examine another receiver whose optimal analog precoder is given by $v_{j,i}^{*}=a_{T}\left(\phi_{k,u,1},\theta_{j,i,1}\right)$ with $\phi_{j,i,1}=\pi /2$ and $\theta_{j,i,1}=\pi /4$. The complex path gains are $\alpha_{k,u,1}=\alpha_{k,u,2}=1$.

Figure A1 shows that $I_{1}$ is much less than 1.0, which confirms that the desired signal in Equation (A1) can be well approximated by $|w_{k,u}^{*H}a_{R}\left(\phi_{k,u,1},\theta_{k,u,1}\right)a_{T}^{H}\left(\phi_{k,u,1},\theta_{k,u,1}\right)v_{k,u}^{*}{|}^{2}$. Similarly, from Figure A2, it can be seen that the second term $I_{3}$ is much smaller than $I_{2}$. Thus, the interference term can be approximated by $|w_{j,i}^{*H}a_{R}\left(\phi_{k,u,1},\theta_{k,u,1}\right)a_{T}^{H}\left(\phi_{k,u,1},\theta_{k,u,1}\right)v_{j,i}^{*}{|}^{2}$.

## Appendix C. The Derivation for Equation (39)

From Proposition 1, *Z* can be approximated as

(A5) $$\begin{array}{cc} Z & =\max|w_{k,u}^{*H}H_{k,u}v_{k,u}^{*}{|}^{2},\\ \; & \approx \max|D_{k,u}\alpha_{k,u,l}w_{k,u}^{*H}a_{R}\left(\theta_{k,u,l}\right)a_{T}^{H}\left(\theta_{k,u,l}\right)v_{k,u}^{*}{|}^{2},\\ \; & =\max_{0<l<L_{k,u}}{\left|D_{k,u}\alpha_{k,u,l}\right|}^{2}. \end{array}$$

Since $\alpha_{k,u,l}$ has the distribution of $X^{2}\left(2\right)$, the CDF of Z can be derived in a straightforward manner [31]:

(A6) $$F_{Z}\left(z\right)={\left(1-e^{-\frac{z}{C}}\right)}^{L_{k,u}}.$$

## Appendix D. The Derivation for Equation (43)

Recalling Equation (35), the SINR can be represented as

(A7) $$X=\frac{Z}{1/\gamma +Y}.$$

To derive the CDF of SINR, we must first calculate the CDF of *Y*. From Proposition 1, *Y* can be represented as

(A8) $$\begin{array}{cc} Y\approx & ZE\left[\sum_{j\neq k}^{K}{\left|v_{k,u}^{*H}V_{j}\right|}^{2}+\sum_{t\neq u}^{M_{k}}{\left|v_{k,u}^{*H}v_{k,t}\right|}^{2}\right],\\ \approx & Z\left(N_{U}-1\right)E\left[{\left|v_{k,u}^{*H}v_{j,i}\right|}^{2}\right]. \end{array}$$

As the AoDs and AoAs are i.i.d for UPA antennas, the expectation can be calculated as

(A9) $$\begin{array}{cc} \; & E\left[{\left|v_{k,u}^{*H}v_{j,i}\right|}^{2}\right]\overset{▵}{=}T\\ = & \frac{1}{4\pi^{2}N_{T}^{2}}\int_{0}^{2\pi }\cdots \int_{0}^{2\pi }{\left|B(\theta_{k,u}\theta_{j,i}\phi_{k,u}\phi_{j,i})\right|}^{2}d\theta_{k,u}\cdots d\phi_{j,i}, \end{array}$$

where B(·) is given by

(A10) $$\begin{array}{cc} \; & B(\theta_{k,u}\theta_{j,i}\phi_{k,u}\phi_{j,i})\\ = & \sum_{q=0}^{Q-1}\sum_{p=0}^{P-1}e^{j\kappa d\left[p\left(\sin\phi_{k,u}\sin\theta_{k,u}-\sin\phi_{j,i}\sin\theta_{j,i}\right)+q\left(\cos\theta_{k,u}-\cos\theta_{j,i}\right)\right]},\\ = & \frac{{\left(1-e^{j\kappa dn\left(\sin\phi_{k,u}\sin\theta_{k,u}-\sin\phi_{j,i}\sin\theta_{j,i}\right)}\right)}^{P}}{1-e^{j\kappa dn\left(\sin\phi_{k,u}\sin\theta_{k,u}-\sin\phi_{j,i}\sin\theta_{j,i}\right)}}\times \frac{{\left(1-e^{j\kappa dn\left(\cos\theta_{k,u}-\cos\theta_{j,i}\right)}\right)}^{Q}}{1-e^{j\kappa dn\left(\cos\theta_{k,u}-\cos\theta_{j,i}\right)}}. \end{array}$$

As shown in Figure A3, the value of *T* can be numerically estimated. As the number of antennas increases, the value of *T* decreases gradually, which confirms Equation (20).

For a given CDF of *Z* in Equation (39), the CDF of SINR can be computed as

(A11) $$\begin{array}{ccc} F_{X}\left(x\right) & = & P(X\leq x)\\ & = & P\left(\frac{Z}{1/\gamma +Z(N_{U}-1)T}\leq x\right),\\ & = & P\left(Z\leq \frac{x}{\gamma (1-x\left(N_{U}-1\right)T)}\right),\\ & = & {\left(1-e^{-\frac{x}{C\gamma (1-T\left(N_{U}-1\right)x)}}\right)}^{L_{k,u}}, \end{array}$$

where we have γ>0 in the derivation above.

## Appendix E. Proof for Equation (48)

Suppose f(p) is a concave function on a convex neighborhood C and differentiable at p. Then, for every y∈C, we have the following inequality based on the definition of concavity:

(A12) $$\begin{array}{cc} \; & f((1-\lambda )p+\lambda y),\\ = & f\left(p+\lambda (y-p)\right),\\ \geq & f\left(p\right)+\lambda \left(f(y)-f(p)\right), \end{array}$$

where 0<λ≤1.

Rearranging the terms and dividing both sides by λ, we have

(A13) $$\frac{f\left(p+\lambda (y-p)\right)-f\left(p\right)}{\lambda }\geq f\left(y\right)-f\left(p\right).$$

Letting λ→0, it can be shown that the left hand side of Inequality (A13) converges to $f^{\prime }\left(p\right)\cdot \left(y-p\right)$. Finally, we have Equation (48) as:

(A14) $$f\left(p\right)+f^{\prime }\left(p\right)\cdot \left(y-p\right)\geq f\left(y\right).$$
